# Epidemiology of invasive fungal infections in the intensive care unit: results of a multicenter Italian survey (AURORA Project)

**DOI:** 10.1007/s15010-013-0432-0

**Published:** 2013-03-06

**Authors:** M. T. Montagna, G. Caggiano, G. Lovero, O. De Giglio, C. Coretti, T. Cuna, R. Iatta, M. Giglio, L. Dalfino, F. Bruno, F. Puntillo

**Affiliations:** 1 Department of Biomedical Science and Human Oncology—Hygiene Section, University of Bari Aldo Moro, Piazza G. Cesare 11, 70124 Bari, Italy; 2 Department of Emergency and Organ Transplantation—Anesthesia and Intensive Care Unit, University of Bari Aldo Moro, Piazza G. Cesare 11, 70124 Bari, Italy

**Keywords:** Intensive care unit, Invasive fungal infection, Candidemia, Drug susceptibility

## Abstract

**Purpose:**

The aims of this study are to evaluate the epidemiology of invasive fungal infections (IFIs) in patients admitted to an intensive care unit (ICU) in Southern Italy and the in vitro antifungal susceptibility of isolates.

**Methods:**

A surveillance program was implemented in 18 ICUs. IFI cases were recorded using a standardized form.

**Results:**

A total of 105 episodes of IFIs occurred in 5,561 patients during the 18-month study. The main infections were caused by yeasts, more than filamentous fungi (overall incidence of 16.5 cases per 1,000 admissions and 2.3 cases per 1,000 admissions, respectively). The overall crude mortality rate was high (42.8 %), particularly for mold infections (61.5 %). All yeast infections were *Candida* bloodstream infections. Over half (59.8 %) were caused by *Candida* non-*albicans*, with *C. parapsilosis* being the most common (61.8 %). In the multivariate model, trauma admission diagnosis, prolonged stay in the ICU, and parenteral nutrition were independently associated with candidemia due to *C. parapsilosis* [odds ratio (OR) 3.5, (1.8–5.2); OR 3.5, (1.02–3.5); OR 3.6, (1.28–6.99), respectively]. Among mold infections, 12 patients suffered from invasive pulmonary aspergillosis, with *Aspergillus fumigatus* as the predominant pathogen (41.7 %). One case of brain scedosporiosis was identified. Overall, azoles and echinocandins resistance was uncommon.

**Conclusions:**

*Candida* non-*albicans* species are the most frequent cause of candidemia in ICU patients. Mold infections are associated with a high mortality rate. This study confirms the importance of the epidemiological surveillance on IFIs in the ICU setting for documenting species distribution and antimicrobial susceptibility patterns to guide therapeutic choices.

## Introduction

The incidence of invasive fungal infections (IFIs) has increased significantly worldwide, representing an important infective complication in hospitalized patients. In particular, critically ill patients are highly susceptible to IFIs: these diseases are very worrisome in the intensive care unit (ICU) due to the complexity of the patients’ underlying conditions.

The epidemiology of IFIs is generally characterized by geographical and temporal variability. Different incidence rates and new emerging species have been revealed during the last 20 years: the European data estimate an incidence of *Candida* bloodstream infection (BSI) ranging between 6.7 and 54 per 1,000 ICU admissions, with a mortality of 33.9–61.8 % [1, 2]. Until recently, *Candida albicans* was the most prominent species in ICU patients [3]. However, a shift towards *Candida* non-*albicans* (CnA) throughout the world has been reported: *C. glabrata* accounts for around 20 % of ICU-associated BSI in some areas [1, 4, 5], while *C. parapsilosis* is the most relevant non-*albicans* species elsewhere [6–8].

Recently, also, invasive aspergillosis has gained importance in non-neutropenic critically ill patients [9, 10], while IFIs caused by other filamentous fungi (i.e., fusariosis, mucormycosis, scedosporiosis) are rare in this setting [11].

Due to the poor outcome related to IFIs in critically ill patients, the knowledge of local epidemiologic trends and antifungal susceptibility of etiological agents is critical. We aimed to determine the contemporary epidemiology, management, and outcome of IFIs in ICU patients in Southern Italy, as well as the in vitro antifungal susceptibility of isolates.

## Materials and methods

### Design of the study

This study is a subset of the AURORA Project [12, 13], a multicenter, observational study, performed between February 2007 and August 2008.

A surveillance program was implemented in 18 ICUs of 16 hospitals. All consecutive adult patients (≥18 years old) who had a documented IFI, either on admission or during stay, were enrolled. The Institutional Review Board approved the protocol. Informed consent was obtained from patients or their representative.

In order to standardize clinical recruitment and microbiological procedures, a training course was performed before starting the study, and a manual was distributed to all participants. In addition, a *memento* was carried out monthly by e-mail or by phone, to both microbiologists and clinicians, to estimate the fullness of reporting and to ensure that all cases were notified. According to protocol, each hospital-associated microbiology laboratory carried out a mycological analysis on biological samples from potentially infected sites, and all fungal species were isolated, identified, and stored at −80 °C. The galactomannan (GM) antigen test was performed in patients with clinical signs and/or symptoms suggestive of invasive aspergillosis (e.g., development of pulmonary infiltrates on chest X-ray, fever refractory to at least 3 days of appropriate antibiotics, pleuritic chest pain).

For each IFI case, the participating center had to complete an electronic report form including demographic and clinical data [age, gender, comorbidities, ICU admission typology, severity of illness on ICU admission, organ dysfunctions on enrollment, length of stay, presence of central venous catheter (CVC) and its removal or not on IFI diagnosis, steroid therapy, total parenteral nutrition, mechanical ventilation, concomitant bacterial infections and antibiotic therapy, antifungal prophylaxis and treatment], microbiological data (e.g., date of first positive culture, etiological agents, fungal colonization), radiological and/or histological findings, and outcome. In order to confirm the identification of fungal isolates, test their in vitro antifungal susceptibility, and analyze the data, both the isolates and the electronic reports were sent to the Coordinating Centre (Laboratory of Mycology—Department of Biomedical Science and Human Oncology, University of Bari Aldo Moro, Bari, Italy).

### Definitions

Yeast IFI was defined as the recovery of yeast from blood culture or other normally sterile site. Fungemia was considered to be catheter-related if a catheter tip culture yielded the same yeast isolated in the bloodstream. Persistent fungemia was defined as the persistence of positive blood cultures for >2 days from the time of the first positive blood culture. *Candida* colonization was defined as repeated growth of yeasts from at least two different non-sterile sites. According to Pittet et al.’s definitions [14], the colonization index (CI) was defined as the ratio of the number of distinct non-blood body sites colonized by *Candida* to the total number of body sites cultured. Patients with a CI ≥0.5 were considered to have multifocal colonization.

Mold IFI was defined as filamentous fungi (e.g., *Aspergillus*, *Fusarium*, *Mucormycetes*) isolation from a normally sterile or non-sterile body site, in conjunction with suggestive clinical manifestations and instrumental test findings.

Corticosteroid treatment was defined as exposure to ≥10 mg/day prednisone equivalent for ≥30 days. Severity on admission was defined by the APACHE II score and organ dysfunction on IFI diagnosis was computed by the Sequential Organ Failure Assessment (SOFA) score [15]. Outcome was defined as survival or death within 30 or 60 days from the incident episode, in yeast and mold IFIs, respectively.

### Laboratory procedures

All biological samples were cultured on two Sabouraud chloramphenicol dextrose agar plates (bioMèrieux, Marcy l’Etoile, France), incubated at 36 ± 1 and 28 °C (for yeasts and molds isolation), and examined daily until 15 days.

Yeasts identification was performed with sugar assimilation profiles obtained using the ID32C kit (bioMérieux, Marcy l’Etoile, France). Filamentous fungi were identified at the levels of genera and species using macro- and micromorphology observations, according to standard methods [16].

GM in serum and bronchoalveolar lavage (BAL) was measured using a sandwich ELISA (Platelia *Aspergillus* Ag, Bio-Rad, Marnes La Coquette, France). An optical density ratio ≥0.5 in serum and ≥1 in BAL was considered to be positive. Samples that yielded positive results, in which interference was known to have occurred, were excluded.

Antifungal susceptibility tests were performed for yeast and mold isolates. The following drugs were supplied by the manufacturers as pure standard compounds: anidulafungin (AND), fluconazole (FLC), and voriconazole (VRC) (Pfizer Pharmaceuticals, Groton, CT, USA); caspofungin (CSP) and posaconazole (PSC) (Merck & Co., Inc., Whitehouse Station, NJ, USA); amphotericin B (AmB) (Sigma-Aldrich, Milan, Italy). The antifungal susceptibility was evaluated by broth microdilution assay performed according to the methodology recommended by the Clinical and Laboratory Standards Institute (CLSI) [17, 18]. *C. parapsilosis* ATCC 22019, *C. krusei* ATCC 6258, *Aspergillus fumigatus* ATCC 204305, and *A. flavus* ATCC 204304 were used as quality controls and tested in each run of the experiments.

For *Candida* spp., the susceptibilities were interpreted taking into account the new species-specific clinical breakpoint suggested by the CLSI subcommittee [19].

AND and CSP minimum inhibitory concentration (MIC) values of ≤0.25 μg/mL were considered to be susceptible (S) for *C. albicans*, *C. tropicalis*; MIC results of ≤2 μg/mL were categorized as S for *C. parapsilosis* and *C. guilliermondii*; AND and CSP MIC end points ≤0.12 μg/mL were considered to be S for *C. glabrata*. *C. albicans* and *C. tropicalis* strains for which the echinocandin MIC was ≥1 μg/mL (≥0.5 μg/mL for *C. glabrata*) are considered to be resistant (R). *C. parapsilosis* and *C. guilliermondii* strains for which the echinocandin MIC resulted ≥8 μg/mL were considered to be R.

FLC MIC end points ≤2, 4, and ≥8 μg/mL were categorized as S, susceptible dose-dependent (SDD), and R for *C. albicans*, *C. tropicalis*, and *C. parapsilosis*. FLC MIC values ≤32 and ≥64 μg/mL were considered to be SDD and R for *C. glabrata*.

For VRC, *C. albicans*, *C. tropicalis*, and *C. parapsilosis* were categorized as S in cases of MIC ≤0.125 μg/mL, intermediate (I) in cases of MIC 0.25–0.5 μg/mL, and R in cases of MIC ≥1 μg/mL. The epidemiological cut-off value (ECV) ≥1 μg/mL was used to detect resistance in *C. glabrata* [20]. The species-specific breakpoint for VRC was used for PSC.

Regarding AmB, in accordance to the literature data [21], a breakpoint ≤1.0 μg/mL was selected to define the isolates as S.

Clinical breakpoints have not yet been established for any antifungal agent against *Aspergillus* spp.; however, we applied ECVs suggested by Espinel-Ingroff et al. [22–24] to examine the susceptibility of this mold to the antifungal agents.

### Statistical analysis

Continuous normally distributed data are expressed as mean and standard deviation (SD) and compared using unpaired Student’s *t*-test. Non-normally distributed data are expressed as median and interquartile ranges (IQR) and compared using the Mann–Whitney *U*-test. Categorical data are expressed as number and percentage and compared using χ^2^ or the Fisher’s exact tests. Logistic regression analysis was then performed, with *Candida* species as the dependent variable. Variables with a *p*-value <0.10 in the univariate analysis and those judged to be clinically relevant were included in the model. The power of the model was tested by the Hosmer–Lemeshow goodness-of-fit test. The effect of potential confounding factors was determined by introducing each factor independently in the final model and considering the variation in the model fit [25]. To evaluate the role of each variable as an independent risk factor, all variables associated with *Candida* species at the <0.05 level of risk in the logistic analysis were introduced in a backward stepwise logistic regression model with an α to remove of 0.05. In all comparisons, a *p* < 0.05 was considered to be statistically significant. Data analysis was performed by using the Statistical Package for the Social Sciences (SPSS) software.

## Results

Among 5,561 patients consecutively admitted to the participant ICUs during the 18-month survey, 105 patients with IFI were identified. The overall IFI incidence was 18.9 cases per 1,000 admissions. Yeasts and molds were responsible for 87.6 and 12.4 % of cases, respectively. Overall, the most common predisposing factor for IFI was CVC presence (94.3 %), followed by total parenteral nutrition (84.8 %), mechanical ventilation (82.9 %), ICU stay >7 days (76.2 %), and antibiotic therapy (73.3 %).

The median age of the patients was 60 (44.5–71) years, with 63.8 % being males and 36.2 % females. Patients older than 60 years accounted for 47.6 % of cases. The admitting diagnosis was medical in 41 %, surgical in 33.3 % (57 % intra-abdominal), and trauma in 25.7 % of patients. The median SOFA score on enrollment was 7 (6–8). In 11 cases (10.5 %), IFI was already present on ICU admission, while in 94 episodes (89.5 %), it occurred during ICU stay, with an onset time of 33.4 (± 25.0) days.

No statistically significant difference in age, gender, previous hospitalization, length of stay (LOS), preceding IFI onset, SOFA score, and antifungal prophylaxis was detected between mold or yeast IFIs. As compared to yeast IFIs, mold IFIs presented a higher frequency of comorbidities, steroid therapy, hematological malignancy, and medical pathology as the admission diagnosis (*p* < 0.05) (Table 1).

**Table 1:** Patient characteristics of 105 patients with invasive fungal infection (IFI) in the intensive care unit (ICU)

Characteristics	Yeast infection, *n* = 92	Mold infection, *n* = 13
Age (years), median (IQR)	60 (43.25–61.75)	59.5 (48.25–69)
Male sex, *n* (%)	58 (63.0)	9 (69.2)
Comorbidities, *n* (%)	17 (18.5)	11 (84.6)*
COPD	6	6
Diabetes	11	5
Hematological malignancy, *n* (%)	0	5 (38.5)*
Solid tumor, *n* (%)	14 (15.2)	3 (23.1)
Steroid therapy, *n* (%)	15 (16.3)	10 (76.9)*
Previous hospitalization, *n* (%)	33 (35.9)	7 (53.8)
Admission typology *n* (%)		
Surgical pathology	33 (35.9)	2 (15.4)
Medical pathology	33 (35.9)	10 (76.9)*
Trauma	26 (28.2)	1 (7.7)
Pre-IFI diagnosis LOS (days), median (IQR)	20 (10–39.25)	25 (16.25–41.75)
SOFA score on IFI diagnosis, median (IQR)	7 (6–8)	7 (6–8)
Antifungals, *n* (%)		
Prophylaxis	18 (19.6)	0
Empirical	16 (17.4)	0
Mortality (end of follow up), *n* (%)	37 (40.2)	8 (61.5)

*IQR* interquartile range, *COPD* chronic obstructive pulmonary disease, *LOS* length of stay, *SOFA* Sequential Organ Failure Assessment

* Statistically significant *p*-value (<0.05)

### Yeast infections

All 92 yeast IFIs were *Candida* BSI, with 37 (40.2 %) caused by *C. albicans* and 55 (59.8 %) by CnA. Candidemia incidence was 16.5 cases per 1,000 admissions. Among CnA, *C. parapsilosis* was found in 34 cases (61.8 %), followed by *C. glabrata* (16.4 %), *C. tropicalis* (16.4 %), *C. guilliermondii*, *C. intermedia*, and *C. norvegensis* (one each, 1.8 %).

A multifocal colonization was documented in 38 (41.3 %) candidemic patients. In these patients, colonization by the same yeast species isolated from blood was more frequently associated with *C. albicans* (60.5 %) than with CnA (39.4 %). Catheter removal was possible only in 59/89 patients (66.3 %) immediately after the onset of candidemia, and tip culture was performed in 44 cases (74.6 %). In all the tested cases, candidemia was found to be catheter related.

The mean duration of candidemia was 3.8 (±4.0) days. Persistent candidemia was found in 47 patients (51.1 %), mainly in surgical patients (36.2 %). Mixed infection (yeasts and bacteria) was documented in 29 (31.5 %) subjects, generally caused by Gram-negative bacteria (58.6 %), with *Pseudomonas aeruginosa* being the predominant microorganism (37.9 %).

As compared with patients with CnA BSI (Table 2), those with *C. albicans* BSI presented a higher incidence of comorbidities, specifically of diabetes mellitus, and were less frequently exposed to antifungal prophylaxis and to parenteral nutrition. Moreover, *C. albicans* BSI occurred earlier, but antifungal treatment was started later, with respect to CnA candidemic patients. In the logistic model, diabetes mellitus and abdominal surgery were significantly associated with *C. albicans* BSI (Hosmer–Lemeshow goodness-of-fit test, χ^2^ 11.35, *p* = 0.91). In the stepwise analysis, diabetes mellitus was the only independent predictor of *C. albicans* BSI acquisition.

**Table 2:** Demographic and clinical characteristics, and predisposing risk factors associated with bloodstream infections (BSI) due to *Candida albicans* and *C. parapsilosis*

Characteristics	Univariate analysis		Regression analysis		Univariate analysis		Regression analysis	
	*C. albicans* (*n* = 37)	*Candida* non-*albicans* (*n* = 55)	Binomial OR (95 % CI)	Stepwise *p*-value	*C. parapsilosis* (*n* = 34)	*Candida* non-*parapsilosis* (*n* = 58)	Binomial OR (95 % CI)	Stepwise *p*-value
Age (years), median (IQR)	58 (41–71)	60 (44–73)			58 (42.75–70)	61 (49.5–63.25)		
Male sex, *n* (%)	21 (56.7)	37 (67.3)			23 (67.6)	35 (60.3)		
Comorbidities, *n* (%)	14 (37.8)	9 (16.3)*			4 (11.7)	19 (32.7)*		
Diabetes mellitus	8 (21.6)	3 (5)*	4.87 (1.02–9.3)*	0.023	2 (5.5)	9 (15.5)		
Steroids, *n* (%)	8 (21.6)	7 (12.7)			5 (14.7)	10 (17.2)		
Solid tumor, *n* (%)	6 (16.2)	8 (14.5)			5 (14.7)	9 (15.5)		
Previous hospitalization, *n* (%)	17 (46)	16 (29)			9 (26.5)	24 (41.4)		
Pre-IFI diagnosis LOS (days), median (IQR)	15 (4.5–30.5)	27 (13–43)*			32 (18.25–50.5)	14.5 (5–32.25)*	2.4 (1.02–3.5)*	0.028
Admission typology, *n* (%)								
Surgical pathology	15 (40.5)	18 (32.7)			11 (32.3)	22 (37.9)		
Medical pathology	12 (32.4)	21 (38.2)			9 (26.5)	24 (41.4)		
Trauma	10 (27)	16 (29)			14 (41.2)	12 (20.7)*	3.5 (1.8–5.2)*	0.035
Abdominal surgery	11 (29.7)	8 (14.5)	2.35 (1.87–3.2)*		4 (11.7)	15 (25.8)		
SOFA score on IFI diagnosis, median (IQR)	7 (6-8)	7 (6–8)			6 (5–8)	8 (6–9)		
Antifungal prophylaxis, *n* (%)	3 (8)	15 (27.3)*	0.19 (0.04–0.86)*		3 (8.8)	15 (25.8)	0.24 (0.05–0.88)*	
CVC removal, *n* (%)	21 (56.7)	38 (70)			23 (67.6)	36 (62)		
Parenteral nutrition, *n* (%)	30 (81)	54 (98.2)*			32 (94)	50 (86)*	3.59 (1.28–6.99)*	0.022
Previous antibiotic therapy, *n* (%)	28 (75.7)	42 (76.3)			27 (79.4)	43 (74)		
Mechanical ventilation, *n* (%)	33 (89.2)	43 (78.2)			27 (79.4)	49 (84.5)		
Empirical therapy, median (IQR)	9 (24.3)	7 (12.7)			4 (11.7)	12 (20.7)		
Candidemia duration (days), median (IQR)	1 (1–4)	1 (1–5)			1 (1–5.25)	1 (1–5)		
Concomitant bacteremia, *n* (%)	11 (29.7)	18 (32.7)			8 (23.5)	21 (36.2)		
ΔΤ candidemia therapy, median (IQR)	2 (1–3)	0 (0–1)			2 (2–3.5)	2 (1–5)		
Mortality (end of follow up), *n* (%)	15 (40.5)	22 (40)			9 (26.5)	28 (48.3)*		

*IQR* interquartile range, *LOS* length of stay, *SOFA* Sequential Organ Failure Assessment, *CVC* central venous catheter

* Statistically significant *p*-value (<0.05)

When compared to patients with *Candida* non-*parapsilosis* candidemia (Table 2), the cases of *C. parapsilosis* BSI presented less comorbidities, were more frequently admitted for trauma, and were exposed less frequently to antifungal prophylaxis and more frequently to parenteral nutrition. Moreover, these subjects manifested candidemia later during their ICU stay. In the binomial analysis, trauma admission diagnosis, length of ICU stay, and parenteral nutrition were promoting factors of *C. parapsilosis* candidemia, whereas antifungal prophylaxis manifested a protective role on its occurrence (Hosmer–Lemeshow goodness-of-fit test, χ^2^ 10.52, *p* = 0.127). When these factors were fitted in the stepwise regression model, trauma, ICU LOS, and parenteral nutrition reached statistical significance, being the best independent promoting factors of *C. parapsilosis* BSI.

### Mold infections

Thirteen mold IFIs (12.4 %) were identified, including 12 aspergillosis and one scedosporiosis. The incidence was 2.3 cases per 1,000 admissions. Aspergillosis was diagnosed by *A. fumigatus* isolation from bronchial aspirate or BAL, coupled by the presence of hyphae at direct microscopy, in 5 (41.7 %) patients, and by repeatedly positive GM assays from both BAL and serum in the remaining 7 (58.3 %) patients. In all these patients, the chest X-ray showed suggestive findings (i.e., lobar consolidation, ill-defined nodules).

*Scedosporium apiospermum* was responsible for one brain abscess. Computed tomography (CT) scan and cerebral magnetic resonance imaging (MRI) of this patient revealed an expansive left temporoparietal process with vasogenic edema.

### Antifungal susceptibility testing

All 98 available isolates (92 yeasts, five *A. fumigatus*, and one *S. apiospermum*) were tested for in vitro antifungal susceptibility (Table 3).

**Table 3:** In vitro antifungal susceptibilities (μg/mL) of 98 isolates collected from the 18-month survey, using Clinical and Laboratory Standards Institute (CLSI) methods

Drug	*C. albicans* (*n* = 37)		*C. parapsilosis* (*n* = 34)		*C. glabrata* (*n* = 9)		*C. tropicalis* (*n* = 9)		*C. guilliermondii* (*n* = 1)	*C. intermedia* (*n* = 1)	*C. norvegensis* (*n* = 1)	*A. fumigatus* (*n* = 5)	*S. apiospermum* (*n* = 1)
	Range	MIC_90_	Range	MIC_90_	Range	MIC_90_	Range	MIC_90_	MIC	MIC	MIC	Range	MIC
AmB	0.25–1	1	0.12–2	1	0.5–1	0.5	0.5–2	2	0.25	1	0.5	0.25–0.5	>16
AND	0.008 –2	0.25	0.03–2	2	0.008–1	0.5	0.06–0.5	0.25	1	0.5	0.06	0.03	>16
CSP	0.03–4	0.25	0.25–8	2	0.008–4	0.5	0.25–2	0.12	4	1	1	0.008 –0.06	>16
FLC	0.06–4	0.5	0.06–8	4	4–128	128	0.5–128	128	4	2	0.5	–	–
PSC	0.016–0.25	0.12	0.03–0.5	0.25	0.25–16	16	0.12–8	0.5	0.25	0.03	0.016	0.06–0.12	0.5
VRC	0.008–0.25	0.016	0.008–2	0.5	0.06–4	4	0.03–16	16	0.06	0.016	0.008	0.06–0.25	0.25

*AmB* amphotericin B, *AND* anidulafungin, *CSP* caspofungin, *FLC* fluconazole, *PSC* posaconazole, *VRC* voriconazole

Among yeasts, AmB and CSP had a susceptible rate of 94.6 and 95.5 %, respectively. All *C. parapsilosis* and *C. tropicalis* were AND susceptible, while a resistance was recognized in 2/37 *C. albicans* and 3/9 *C. glabrata* isolates.

All triazoles demonstrated potent activity against *C. albicans* (susceptible rate 100 %) and *C. parapsilosis* (susceptible rate 94.1 %). Regarding *C. glabrata* and *C. tropicalis*, 6/9 and 3/9 isolates resulted susceptible to FLC, respectively, and 4/9 and 2/9 isolates resulted susceptible to VRC, respectively. *C. glabrata* was the least susceptible species to PSC, with an MIC_90_ of 16 μg/mL.

Regarding *A. fumigatus*, AmB (range 0.25–0.5 μg/mL), AND (0.03 μg/mL), CSP (range 0.008–0.06 μg/mL), PSC (range 0.06–0.12 μg/mL), and VRC (range 0.06–0.25 μg/mL) exhibited excellent potency against all five isolates.

*S. apiospermum* had raised MICs of AmB (MIC >16 μg/mL), AND (MIC >16 μg/mL), and CSP (>16 μg/mL), but it was not inhibited by PSC (MIC 0.5 μg/mL) or VRC (MIC 0.25 μg/mL).

### Treatment

No patient with mold IFIs was on antifungal prophylaxis at the time of diagnosis, while 18 (19.6 %) candidemic patients were receiving FLC 200 mg/day, for a mean of 20.6 (±15.4) days. *Candida* spp. isolates from patients on antifungal prophylaxis were less susceptible to FLC, PSC, and VRC (Table 4).

**Table 4:** In vitro azole resistance in the presence/absence of exposure to azole prophylaxis

Azole prophylaxis exposure	Drugs	No. of isolates	No. (%) of isolates resistant^c^
Absence^a^	Fluconazole	72	2 (2.8)
	Voriconazole	72	2 (2.8)
	Posaconazole	72	0
Presence^b^	Fluconazole	17	8 (47.0)
	Voriconazole	17	10 (58.8)
	Posaconazole	17	6 (35.3)

^a^Species included *C. albicans* (34 isolates), *C. parapsilosis* (31), *C. glabrata* (3), and *C. tropicalis* (4)

^b^Species included *C. albicans* (3 isolates), *C. parapsilosis* (3), *C. glabrata* (6), and *C. tropicalis* (5)

^c^Fluconazole resistance was defined as MIC ≥8 μg/mL for *C. albicans*, *C. tropicalis*, and *C. parapsilosis*, and MIC ≥64 μg/mL for *C. glabrata*. Voriconazole resistance was defined as MIC ≥1 μg/mL for *C. albicans*, *C. tropicalis*, and *C. parapsilosis*; an epidemiological MIC cut-off value ≥1 μg/mL was used to detect voriconazole resistance in *C. glabrata*. The resistance species-specific breakpoint for voriconazole was used for posaconazole

Empirical therapy was started in 17.4 % of candidemic patients within 3 (±2) days from symptoms onset. Drugs empirically prescribed were FLC in 62.5 % of cases, followed by AmB (25 %) and CSP (12.5 %). In all cases, empirical therapy was confirmed as the target therapy. Overall, the target antifungal therapy was administered in 98 (91.8 %) patients; the remaining seven patients (five *Candida* spp., one *A. fumigatus*, and one *S. apiospermum* infections) were not treated because of pre-IFI diagnosis death.

Patients with candidemia received most frequently (49.4 %) FLC 400 mg/day, followed by CSP (31 %) 50 mg/day, and AmB (19.6 %) 5 mg/kg/day. Patients with invasive pulmonary aspergillosis (IPA) received VRC 200 mg/day.

### Patient outcome

Overall, the crude mortality rate was 42.8 %. More specifically, yeast cause mortality was 40.2 %. Species-specific mortality ranged from 26.5 % for infection caused by *C. parapsilosis* to 77.8 % by *C. tropicalis*. CVC removal was associated with a better outcome [mortality rate, 63.6 vs. 27.1; odds ratio (OR) 0.21; 95 % confidence interval (CI) 0.08–0.58 *p * < 0.05]; the exclusion of the patients who died within 48 h did not significantly modify this finding.

Autopsy was performed in one patient and showed disseminated candidiasis (kidney and liver).

Among molds, eight patients died (61.5 %); of these, two died (including the scedosporiosis case) as a direct consequence of infection (attributable mortality rate 15.4 %).

## Discussion

Our multicenter survey shows that IFIs represent an important infectious complication in critically ill patients. A high occurrence of cases due to CnA (59.8 %) and a high mold mortality rate (61.5 %) were observed.

The most frequent IFI was *Candida* BSI, with an incidence of 16.5 cases per 1,000 admissions. This finding is higher than the data reported for Northern Europe (6.7 cases to 7.4 per 1,000 admissions) [1, 26] and lower than those reported in other European countries (35.7–54 cases per 1,000 admissions) [2, 27]. In an Italian study, Tortorano et al. [11] found an incidence of 10.08 per 1,000 admissions. The differences among various geographical areas may be due to several factors, including diversity in patient age, variations in clinical and microbiological practices, and differences in drugs usage [28]. CVCs are widely regarded as the most common risk factor for candidemia [3], and their removal is associated with shorter duration of disease and better outcome [29]. In the present study, 96.7 % of patients with candidemia had a catheter in place at the time of diagnosis. We found that all microbiologically evaluated catheters were probably the source of *Candida* BSI, and their removal was associated with higher survival rate (72.9 %).

Although *C. albicans* is still regarded as the most common species [3], recent epidemiologic studies have demonstrated an increasing incidence of CnA candidemia, with *C. glabrata* and *C. parapsilosis* ranked as second in the USA and Northern Europe [4, 5], and in Latin America [30] and Southern Europe [8], respectively. The reason for this change in the pattern of *Candida* species distribution has not yet been completely understood, but some predisposing factors have been identified, such as indwelling catheters and parenteral nutrition for *C. parapsilosis* [7, 31], cancer and neutropenia for *C. tropicalis* [32], and previous exposure to azoles for *C. krusei* and *C. glabrata* [33, 34]. We found that CnA were the most frequent etiologic agents of candidemia, with *C. parapsilosis* ranked first (61.8 %). In agreement with other studies [2, 31], we observed an association between ICU LOS, parenteral nutrition, and *C. parapsilosis* BSI. In reality, *C. parapsilosis* is notorious for its capacity to grow in hyperalimentation solutions with high concentrations of glucose, to form biofilms on catheters and other implanted devices, for nosocomial spread by healthcare workers’ hands, and for its persistence in the hospital environment [31]. These observations suggest the need for more accurate nosocomial surveillance measures, such as hand hygiene, to prevent *C. parapsilosis* BSI and improve the health conditions of patients at risk.

Antifungal resistance was poorly recognized in our study and restricted to a few isolates. According to other authors [35, 36], CSP and AND resistance was low (4.4 % to CSP and 5.6 % to AND). FLC still tended to be quite active against isolates of *C. albicans* and *C. parapsilosis*, while its resistance was mainly associated with *C. glabrata* and *C. tropicalis*. In our study, 8 out of 10 episodes of candidemia caused by FLC-resistant strains (five *C. glabrata* and three *C. tropicalis*) occurred in individuals with previous FLC administration. In addition, higher VRC and PSC MICs tended to be associated with FLC prophylaxis, confirming the potential problem of cross-resistance between azoles [37].

Finally, in our study, the mold infections were less common than yeast infections (ratio 1:7), but accounted for a higher mortality rate (61.5 vs. 40.2 %). Actually, IPA has recently gained importance in the ICU setting, ranging from 0.3 to 6.9 % [9, 38], with an overall mortality rate of 80 % and an attributable mortality of 20 % [39]. It commonly occurs in patients with acute exacerbation of chronic obstructive pulmonary disease, diabetes, and in recipients treated with intravenous corticosteroids [9, 11, 40]. IPA diagnosis is difficult because of non-specific signs and symptoms and due to additional diagnostic examinations often delayed by a poor clinical suspicion [40, 41]. In our study, an IPA incidence of 2.1/1,000 admissions and steroids employed in 76.9 % of these patients were observed. In this setting, the outcome was poor (60 %), in accordance with other reports describing dramatic fatality rates [9, 41].

In conclusion, candidemia is the most frequent IFI in ICU patients (87.6 %), while molds IFI remains a sporadic event (12.4 %). CnA is responsible for over half of the candidemia episodes, with *C. parapsilosis* being the most common (61.8 %). A prolonged ICU LOS, use of invasive procedures (i.e., CVC, parenteral nutrition), and/or inadequate control of fungal infections could explain the high prevalence of *C. parapsilosis*. Mortality remains high (crude mortality 42.8 %), mainly linked to mold infections. Periodic surveillance is necessary in order to estimate the incidence of IFI as well the antifungal drug resistance. Further studies in the ICU will be needed so as to understand more clearly the interaction between fungi and host conditions, to allow earlier and more accurate diagnosis, and minimize the inappropriate use of drugs.
